# Atraumatic fractures of the femur

**DOI:** 10.1259/bjr.20201457

**Published:** 2021-03-18

**Authors:** Ganesh Hedge, Siddharth Thaker, Rajesh Botchu, Richard Fawcett, Harun Gupta

**Affiliations:** 1 Department of Radiology, Royal Lancaster Infirmary, Lancaster, UK; 2 Department of Musculoskeletal Radiology, Leeds Teaching Hospitals, Leeds, UK; 3 Department of Musculoskeletal Radiology, Royal Orthopaedic Hospital, Birmingham, UK

## Abstract

Atraumatic fractures of femur, although not as common as traumatic fractures, are frequently encountered in the clinical practice. They present with non-specific symptoms and can be occult on initial imaging making their diagnosis difficult, sometimes resulting in complications. Overlapping terminologies used to describe these fractures may hamper effective communication between the radiologist and the clinician. In this article, we review various atraumatic fractures of femur, terminologies used to describe them, their imaging findings and differential diagnosis. The article also describes the aetiology, pathophysiology and relevant biomechanics behind these fractures. An approach to atraumatic femoral fractures has been outlined.

## Introduction

Atraumatic femoral fractures are frequently encountered in clinical practice. The femur is the largest weight-bearing bone providing attachments to powerful antigravity muscles and a few of the strongest ligaments and tendons of the body and endures considerable biomechanical forces. In addition to traumatic injuries, the femur is susceptible to fractures even without significant trauma under certain clinical conditions; *e.g.* metabolic bone disease. Atraumatic fractures, contrary to those following trauma, present with vague symptoms, occult on imaging posing challenges during diagnosis. We will describe non-traumatic femoral fractures, their imaging features and differentials in the article. In the end, we will outline to approach these fractures.

### Terminologies

Terminologies used to describe atraumatic fractures are confusing and often overlap.

Atraumatic fracture term donates a fracture caused by a relatively low-energy mechanism that usually considered incapable of producing a fracture.^1^ This broad category includes pathological, stress, fatigue, insufficiency and atypical femoral fractures.

Stress fractures, partial or complete, usually result in bones unable to withstand subthreshold stress applied in a rhythmical and repeated manner.^2^ Such fractures can be of two types: fatigue fractures – from abnormal repetitive stress causing temporal mechanical failure – in a normal and insufficiency fractures – owing to normal stress on an abnormally weakened bone.^3^ The term “Fragility fracture” is exclusive for insufficiency fractures in osteoporosis following single minimally traumatic event.^1,4^

**Figure 1.**
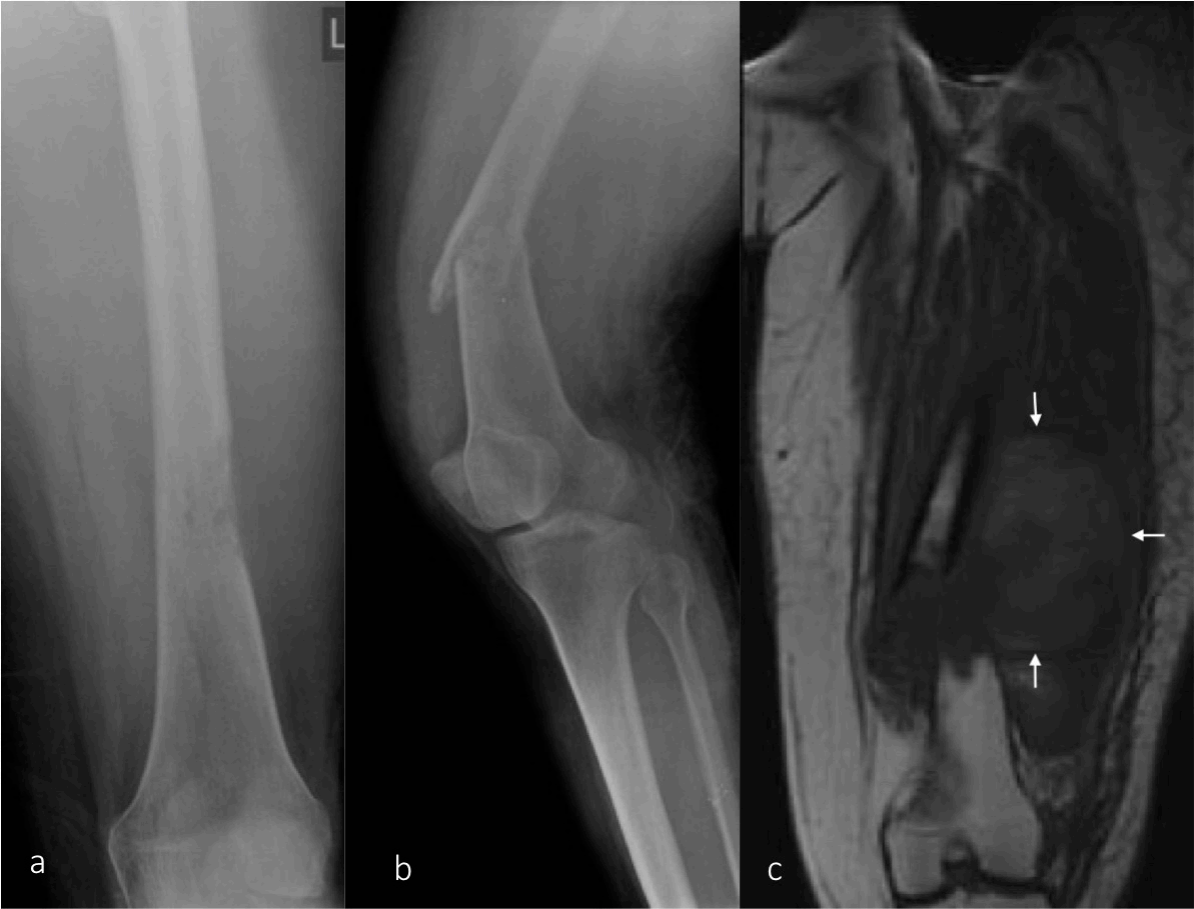
Pathological fracture/ (**a**) AP radiograph of the femur demonstrating a lucent lesion in the distal femur with permeative pattern of bone destruction suggesting an aggressive lesion. (**b**) Lateral view of the knee joint including the distal femur demonstrating a pathological fracture through the lesion. (**c**) T1 coronal MRI image showing aggressive lesion replacing the normal marrow fat at the fracture site with a large adjacent soft tissue component. AP, anteroposterior.

Pathological fractures are insufficiency fractures occurring in a bone weakened by benign or malignant neoplastic lesion affecting its trabecular integrity (Figure 1). By the same definition, fracture through osteomyelitis is regarded as a pathological fracture.^1,5^ (Supplementary Figure 1).

Supplementary Figure 1.

### Fatigue fractures

These are focal fractures in the normal bone due to repetitive stress following sustained microdamage exceeding the bone’s ability to heal employing physiological remodelling. Frequent in young, suboptimally conditioned individuals who abruptly engage in strenuous activities such as weekend-warriors with limited physical fitness, military recruits, and athletes who abruptly intensify their training regimen,^6^ they are also common after orthopaedic surgery in lower limbs, foot in particular where altered gait and osteopaenia following guarded mobility in post-surgical period implied as causative factors.^7^

The femur is the fourth common bone affected by stress injuries accounting for approximately 7.2% cases in athletes.^8^ The femoral neck (50%), the condylar area (24%) and the proximal shaft (18%) are commonly affected anatomical sites and can be bilateral (9% cases).^9^ The medial aspect is at risk and commonly affected due to biomechanical forces exerted on it during weight-bearing and muscle exertion. Compressive forces pass through the medial shaft; whereas, tensile forces act upon the lateral aspect when one bears the weight – the latter is substantially lesser relieved by the iliotibial tract and vastus lateralis action – in contrast of dynamics of vastus medialis, adductor longus and brevis increasing medial compression force.^10,11^

**Figure 2.**
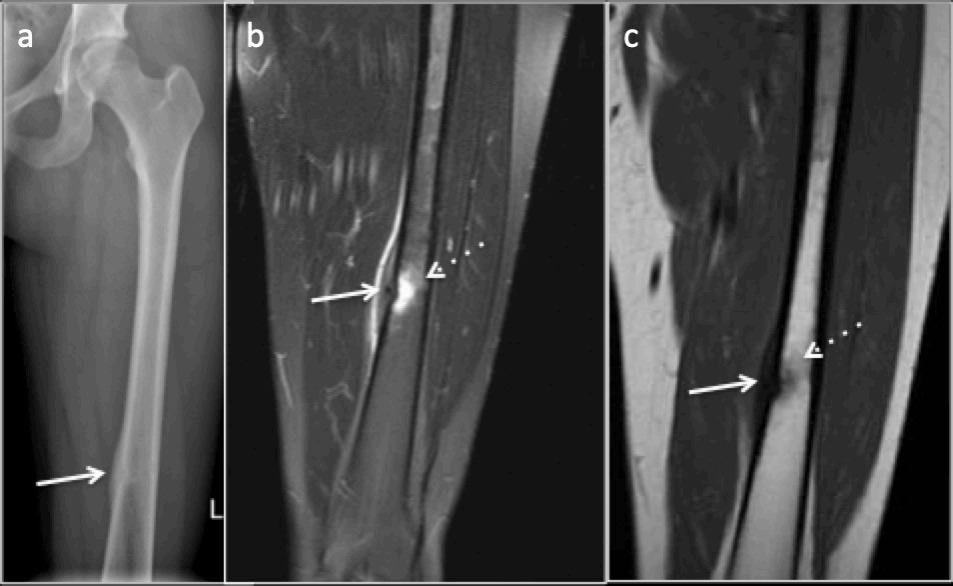
35-year-old female amateur marathon runner with Fatigue fracture in the femoral diaphysis where cortical bone predominates. (**a**) Radiograph demonstrating subtle central lucency with cortical thickening and periosteal reaction (arrow). (**b**) Coronal PDFS image (**c**) Coronal T1 image demonstrating marrow edema (dotted arrow), cortical thickening and periosteal reaction (arrow). PDFS, proton density fat saturated.

Radiographic findings of the fatigue fracture depend on the location of involvement and chronicity of the injury. Radiographically, the earliest finding is a subtle lucency in the cortex often described as “Grey cortex sign”^12^ from microfracture and osteoclastic resorption (Figure 2) followed by periosteal reaction, and cortical thickening on progression because of periosteal and endosteal remodelling and callus formation.^1^ As it lags behind the osteoclastic activity by a few weeks, the periosteal reaction is not visible until several weeks into the disease process.^7^ Cortical breaks may be visible in injuries inciting severe bony responses.

**Figure 3.**
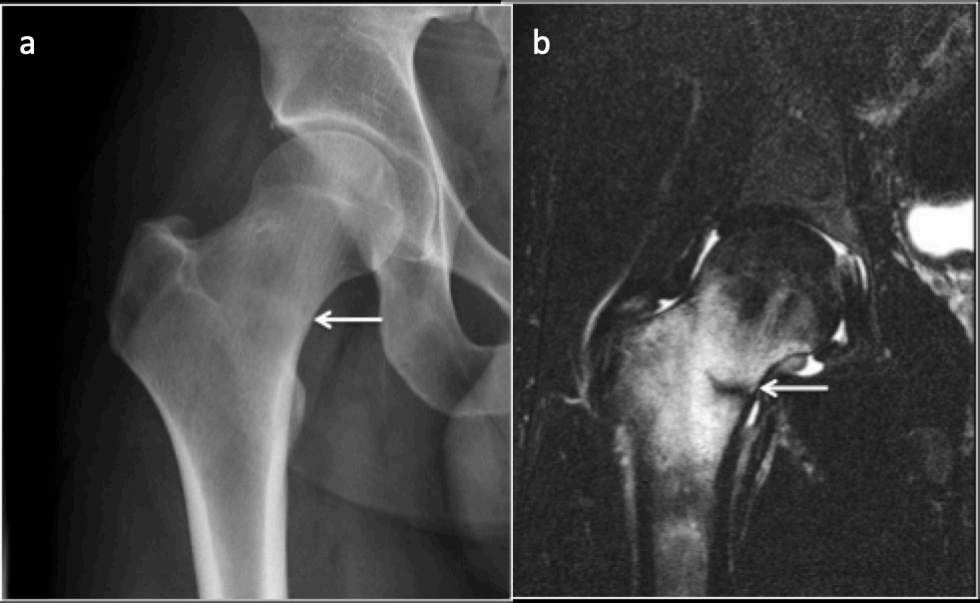
Fatigue fracture in the femoral neck where cancellous bone predominates. (**a**) Radiograph demonstrating subtle intramedullary sclerosis (arrow) along the fracture line with blurred trabeculae. (**b**) Coronal STIR image demonstrating marrow oedema and the linear hypointense fracture line(arrow). STIR, short tau inversion recovery.

Fatigue fractures in femur predominantly affect its cancellous bone dominant parts such as metaphysis, neck and intercondylar region and demonstrate different radiological appearances. The heralding finding is faint blurring and sclerosis of the trabeculae. Following which, linear intramedullary sclerosis due to microcallus becomes evident (Figure 3). For fatigue fractures, MRI is the gold-standard imaging modality with the highest sensitivity and specificity,^13^ however, specificity of MRI may be low for early changes of fracture. Marrow oedema in presence or absence of periosteal reaction is the earliest imaging finding. In more established injuries, a fracture line may be visible appearing as a linear hypointense line on all imaging sequences, usually followed by periosteal and endosteal callus formation.

Proximal femur, particularly intercondylar region, neck and head of femur contain peculiar arrangement of trabeculae, primary compressive trabeculae (vertically oriented), primary tensile trabeculae (more horizontally oriented), secondary compressive trabeculae (obliquely oriented) and greater trochanteric trabeculae. Fractures from compressive stresses and located along the medial aspect of the femur are considered low risk as they tend to reduce with weight bearing and usually managed conservatively. Fractures due to tensile stress, such as fractures along the superolateral aspect of the femoral neck which occur perpendicular to the tensile trabeculae are considered high risk which needs extended rehabilitation, may result in a delayed union or even progress to complete fractures unless treated. Those with a fracture line involving more than 50% of the width of the femoral neck and displaced fractures are also considered high risk and need aggressive management.^1^

Femoral fatigue fractures are typically from compression stress and can be managed conservatively by rest and restricted activities. MRI findings are used to grade the severity of the fatigue fractures and to ascertain return to play or recovery time. (Table 1). According to Arendt grading of stress fractures, time required to return to sports is approximately 3 weeks for Grade 1 injury, 3–6 weeks for Grade 2 injury, 12–16 weeks for Grade 3 injury with Grade 4 injury requiring more than 16 weeks of rest^15^ (Table 2).

**Table 1.** Arendt et al^14^ ^*a*^ grading of the stress fractures

Low grade	
1	Positive signal changes on STIR images only.
2	Positive signal changes on STIR and T2.
High Grade	
3	Positive signal changes on STIR, T1 and T2; no fracture

STIR, short tau inversion recovery.

a Modified with permission from original publication).

**Table 2.** Key features of fatigue fractures

Fatigue fractures are due to repetitive stress, commonly seen athletes or military recruits. Femoral neck is most common location. Radiographic findings are subtle and depend on the location of involvement and chronicity, appearing as subtle lucency in cortical bone and faint blurring and sclerosis of the trabeculae in cancellous bone. MRI is most sensitive and specific imaging modality should be done even when radiographs are negative in appropriate clinical setting. MRI demonstrates marrow oedema and may show fracture line in later stages. MRI classification is useful in assessing the severity of the fracture and return to activity time.

### Insufficiency fractures

#### Subchondral insufficiency fracture

Generally involving elderly females with osteoporosis, subchondral insufficiency fractures (SIF) are infrequent causes of hip pain and can mimic hip osteonecrosis.^15,16^ Bone fragility secondary to osteoporosis is an attributable risk factor exaggerating stress applied immediately below the subchondral area.^17^. SIF, in young individuals, is uncommon^18^ and be seen as fatigue fractures in military recruits,^19^ post-renal and liver transplant^20^ and in patients on oral steroids.^21^ It has also been implicated as a potential precursor of rapidly destructive osteoarthritis.^22^

Radiographs are often unremarkable in initial stages and may show background osteopaenia. A few months later, there may be sclerotic changes due to subchondral fatty saponification and reactive subchondral remodelling. A crescent sign depicting to subchondral collapse is a sign of advanced disease.^18^

**Figure 4.**
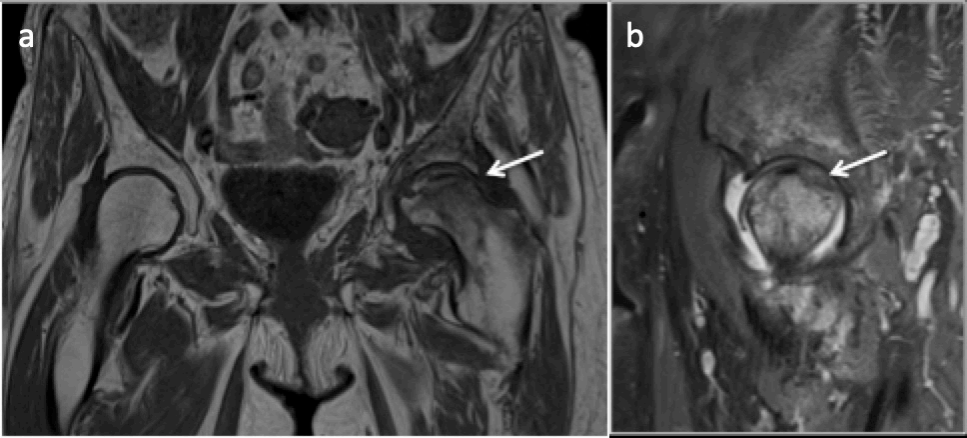
Subchondral insufficiency fracture of the left femoral head. Coronal T1 (a) and Sagittal PDFS (**b**) images demonstrating marrow oedema in the femoral head and neck with Hypo intense irregular fracture line (arrow). PDFS, proton density fat saturated.

**Figure 5.**
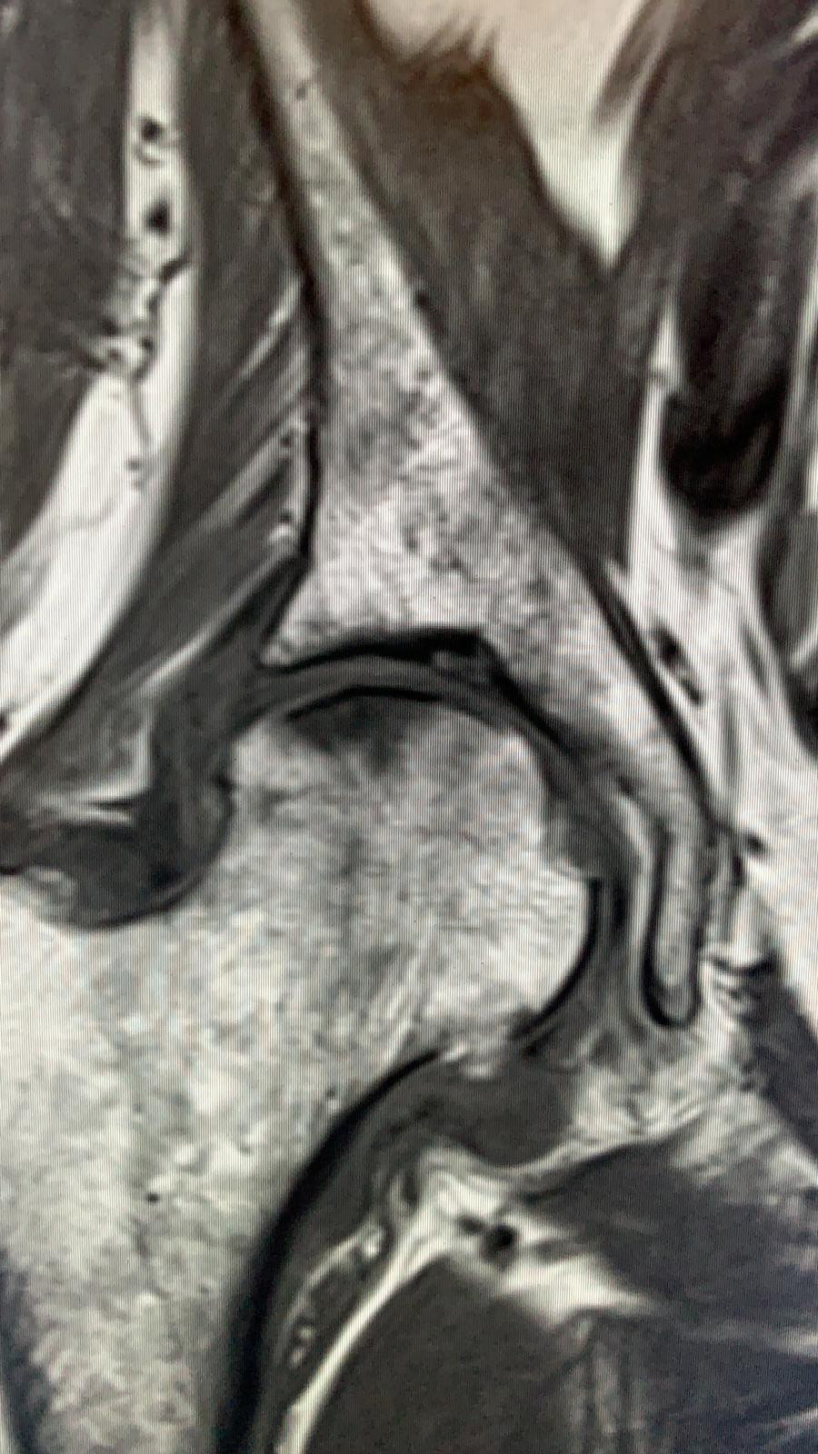
MRI Coronal T1 imaging of right hip demonstrating a linear low signal subchondral line in the superior femoral head consistent with subchondral insufficiency fracture.

MRI findings are characteristic including subchondral-predominant bone marrow oedema on fluid sensitive sequences and a characteristic subchondral low signal intensity (SI) band on T1 images corresponding to fracture line and associated trabecular remodelling^23^ located generally in the superolateral aspect of the femoral head.^23,24^ It is often seen as an irregular, serpiginous, disconnected and convex to the articular surface^23,25^ (Figures 4 and 5). Furthermore, the area between SIF and the articular surface usually demonstrates high signal intensity on *T*_2_ weighted images owing to marrow oedema, especially in the early phase of the fracture.^25^ Prognostically, some SIF can resolve after conservative treatment while others progress to subarticular collapse and subsequent osteoarthritis requiring surgical intervention.^22–24^ According to literature, the length of SIF has prognostic value, being shorter in patients without progression to collapse (mean 13.4 mm) than in patients with progression to collapse (22.5 mm).^26^ The percentage of weight-bearing femoral head involved is also described as a prognostic factor for predicting the progression to collapse.^26^

**Figure 6.**
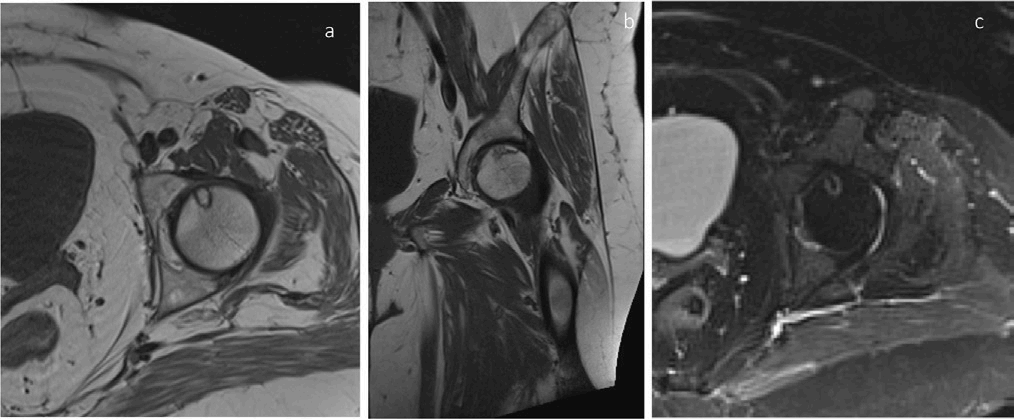
T1 axial (a) and Coronal (b) and T2 fat saturated axial (c) images demonstrating osteonecrosis (avascular necrosis) with a smooth well-circumscribed margin.

Important differential diagnosis of SIF includes femoral head osteonecrosis and transient osteoporosis. In osteonecrosis, previously mentioned low-intensity band representing repair tissue tends to be smooth and circumscribed around necrotic segment entirely^24–26^ (Figure 6) compared to irregular fracture line in SIF. Subchondral collapse is a feature of established osteonecrosis.^26^ In gadolinium-enhanced MRI, SIF demonstrate contrast enhancement due to the presence of viable bone between fracture line and weight-bearing cortex which is absent in osteonecrosis due to nonviable bone.^24^

In transient osteoporosis, bone marrow oedema is seen in femoral head and neck, sometimes sparing the subchondral region^27^ and with the absence of subchondral low SI band (Table 3).

**Table 3.** Key features of subchondral insufficiency fracture

Most commonly seen in elderly osteoporotic females. Radiographs are often unremarkable and MRI is imaging modality of choice. Marrow oedema with irregular, disconnected subchondral fracture line which is convex to articular surface is characteristic MRI finding. Osteonecrosis of femoral head and transient osteoporosis are important differentials.

### Osteomalacia

Osteomalacia is characterised by deficient mineralisation of the osteoid in affected bones. Defective deposition of calcium salts remains a cause due to low serum calcium, phosphorus or both caused either by its impaired absorption from the gastrointestinal tract or increased renal excretion. Low in their mineral content, the weakened bones become too soft and bend readily predisposing them for deformities and fractures.

**Figure 7.**
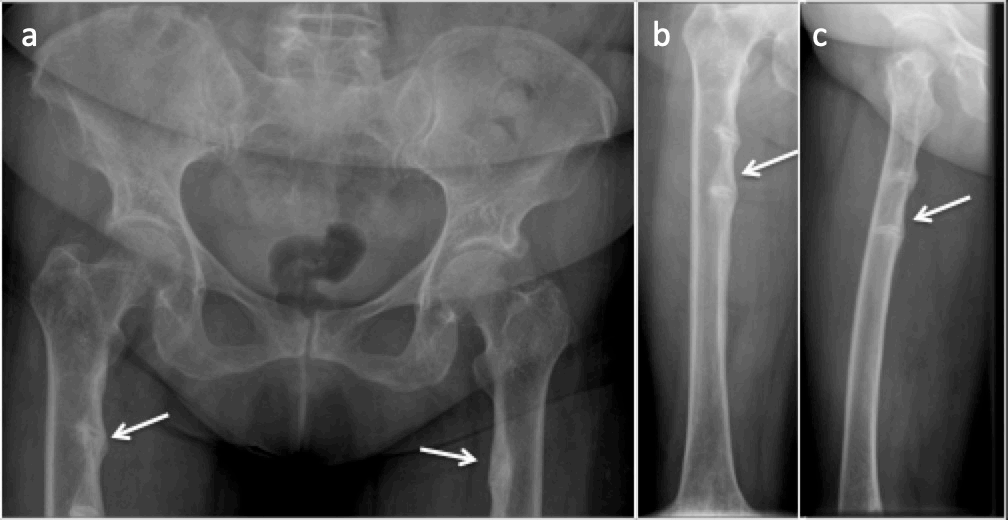
(**a**) Anteroposterior radiograph of the pelvis. (**b**) Anteroposterior and (**c**) Lateral radiograph of the right femur showing looser zones of osteomalacia. Multiple, bilateral lucency which involve only a part of femoral shaft along the medial aspect, oriented perpendicular to the cortex with associated surrounding sclerosis.

It results in reduced bone mass with ill-defined trabecular bone following excessive deposition of the unmineralised osteoid, showing intermediate density on CT between that of bone and marrow.^28^ Looser zones or “Pseudo fractures” are pathognomonic findings of osteomalacia. By definition, they are stress fractures^29^ resulting from deposition of the unmineralised matrix at the site of stress or nutrient vessels.^28^ Manifesting typically late in the disease course, they follow minimal or no trauma. They share similar anatomic locations with stress fractures, usually seen on the medial aspect of the femur as broad lucencies perpendicular to the cortex, often bilateral, symmetrical and sometimes multiple. They demonstrate minimal marginal sclerosis and callus with delayed healing^29,30^ (Figure 7).

### Incremental fractures

The is reserved for insufficiency fractures seen in the Paget’s disease. The aetiology of the Paget’s disease is unknown and is characterised by excessive and abnormal remodelling of bone which results in abnormally enlarged but weakened bone often resulting in affected bowed femur. Fractures are the most common complications^31^ typically involving lateral/convex aspect of the proximal femur.

**Figure 8.**
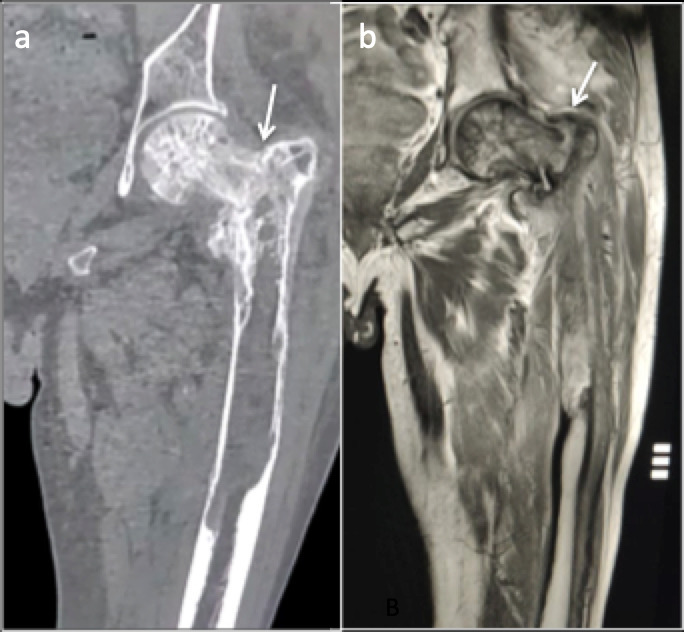
Paget’s disease of left proximal femur with slight bowing change and intertrochanteric fracture (arrows). (**a**) Coronal CT reformat and (**b**) Coronal MRI T1 imaging.

On radiographs, they appear as a single or multiple linear, incomplete, transversely oriented cortical lucencies initially. They may become complete with minimal or no trauma, described as “Banana fracture”. These fractures notoriously show high prevalence of non-union^31,32^ (Figure 8).

### Atypical femoral fractures

Atypical femoral fractures (AFF) are insufficiency fractures occurring in the subtrochanteric location involving the femoral diaphysis. They are strongly linked to long-term therapy for osteoporosis with bisphosphonates^33^ or other antiresorptive medication such as RANKL inhibitors.^34^ Other medications such as long-term glucocorticoid therapy, proton pump inhibitors are also implicated. Some of the other bone diseases such as hypophosphatasia, pyknodysostosis and osteopetrosis, vitamin D deficiency, and rheumatoid arthritis^35^ have also been associated with the occurrence of AFF.

Pathophysiology of the AFF is still poorly understood. Bisphosphonates used in the treatment of osteoporosis act by inhibiting the osteoclasts, thus reducing the bone turnover and resorption. In old age, as bone resorption outweighs the new bone formation, this is a beneficial response resulting in improved bone mass in osteoporosis. However, osteoclast inhibition also results in compromised natural repair capacity of the bone resulting in accumulation of the microdamage over time. Altered bone mineral and matrix composition due to reduced bone turn over results in increased mineral to matrix ratio (hypermineralisation) which are often visible on the radiograph as the thickened bone cortex. Reduced bone turnover also results in accumulation of the advanced glycation end products in the extracellular matrix and increased mean tissue age. These changes, although result in increased bone strength and stiffness, makes them brittle and susceptible to fractures.^35,36^ These brittle bones are more susceptible for tensile stresses which act along the lateral femoral cortex resulting in microdamage, which in turn may progress to fracture along the lateral femoral shaft. It has been described that biomechanical forces may result in micromotions at the early fracture site preventing its healing.^36^ Specific variant geometries in the hip and femur such as varus angle of the femoral neck, narrow centre-edge angle and high BMI have been described to be associated with the development of atypical femur fracture in long-term bisphosphonate users^37^ (Supplementary Figure 2).

Supplementary Figure 2.

In 2013, American Society of Bone and Mineral Research (ASBMR) published a revised set of clinical and imaging criteria to define AFF and to exclude findings that raise the possibility of different causes for the fracture.^35^

**Figure 9.**
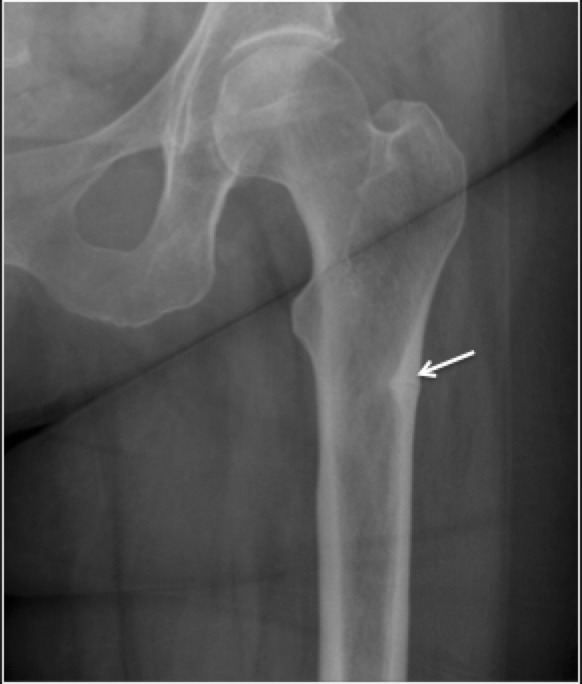
Anteroposterior radiograph of the proximal femur with atypical femoral fracture in the left proximal femur. Note the lucent transverse fracture line with endosteal and periosteal beaking (arrow).

In a patient with bisphosphonate therapy following osteoporosis presenting with hip or thigh pain, careful evaluation of the radiograph should be made to identify the subtle findings. Classical AFF is located along the lateral cortex of femur in the femoral diaphysis with a transverse fracture line occurring with no or minimal trauma. When the fracture line involves periosteum or endosteum, there may be periosteal/endosteal thickening (Figure 9). Focal cortical thickening is also seen at the fracture site. This, along with the transverse orientation of the fracture line is considered as the most sensitive, specific and accurate criteria in diagnosing bisphosphonate-related AFF^38^ and has shown to be useful in differentiating AFF from other fractures involving the lateral cortex such as a Banana fracture in Paget’s disease.^38^

**Figure 10.**
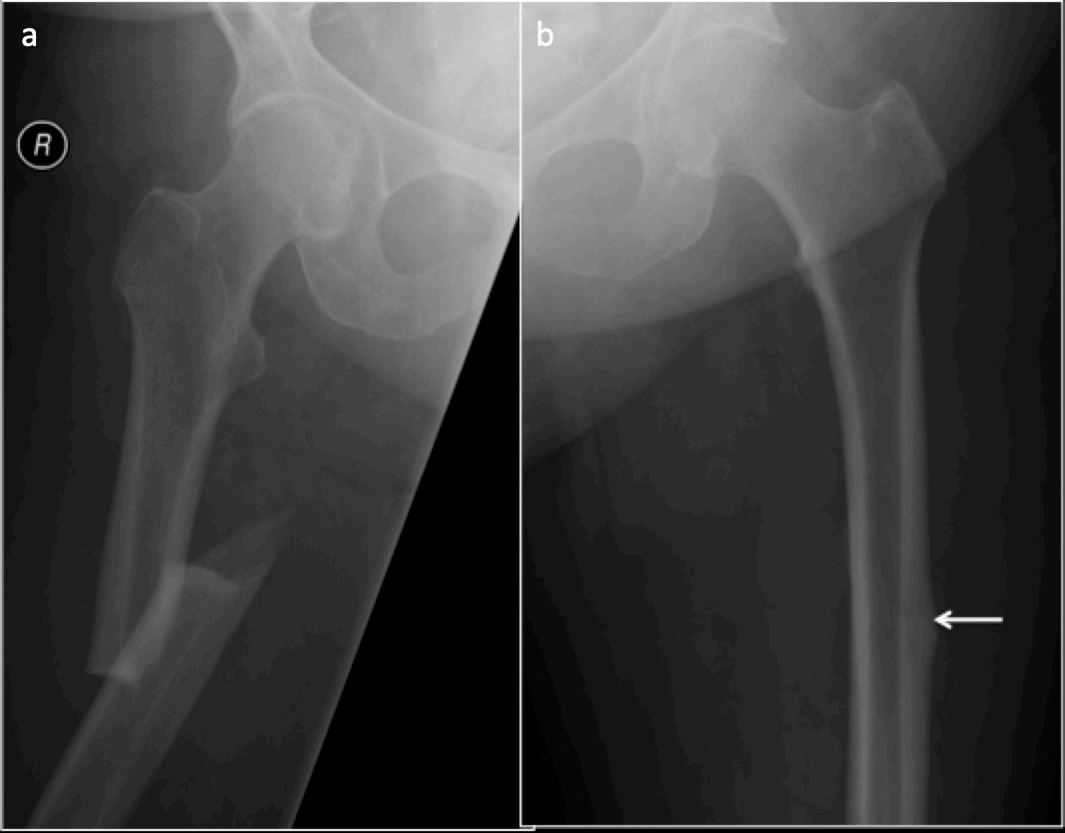
Bilateral involvement of atypical femoral fracture. 73-year-old female on bisphosphonates (**a**) Complete displaced fracture of the right femoral shaft. (**b**) Cortical and periosteal thickening and beaking in the outer cortex of the left femoral shaft indicating early changes of incomplete atypical femoral fracture (arrow).

**Figure 11.**
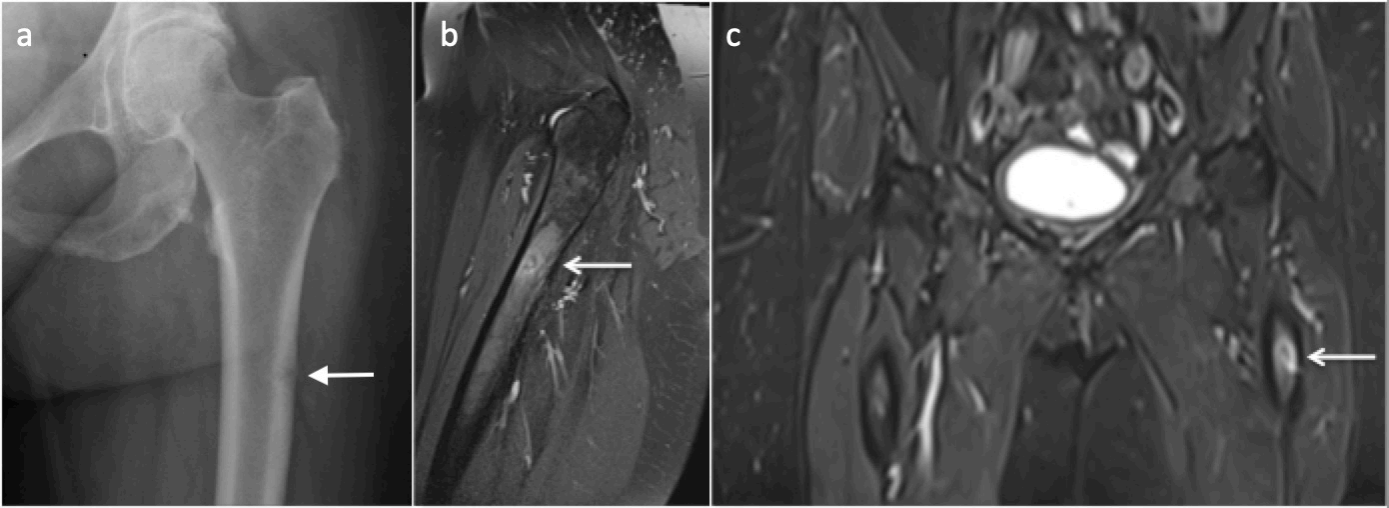
56 year-old-female with hypophosphatasia with atypical femoral fracture. Anteroposterior radiograph of the femur (**a**) demonstrates a lateral diaphysis lucency and MRI PDFS sequences (**b, c**) transverse cortical lucency (arrows) along the lateral femoral cortex. PDFS, proton density fat saturated.

Bilateral involvement has been described in 40–50% of the patients with AFF,^35,39^ thus when an AFF is identified, screening of the contralateral hip and entire femur is recommended (Figures 10 and 11). In case of a normal radiograph of the opposite side, with high clinical suspicion, further imaging with CT or MRI is favoured^36^ (Table 4).

**Table 4.** Key features of atypical femoral fracture

Newly described distinct fracture often seen in osteoporotic patients on long-term. bisphosphonate therapy, however can occur in other patients as well. Classically located along the lateral cortex of the femoral diaphysis. Diagnosis is based on ASBMR criteria. Bilateral involvement is common and screening of contralateral femur is recommended.

### Pathological fractures

The femur is a common site for pathological fractures. Pathological fractures in femur are usually secondary to metastatic lesions and subtrochanteric femur is commonest location. Primary bone tumours are responsible for only 6% of pathological fractures. MRI is a reliable imaging modality to identify and differentiate pathological fracture from non-pathological or stress fracture and T1 images are vital. T2 and fluid sensitive sequences are non-specific due to the presence of excessive marrow oedema and surrounding inflammation.

They can be characterised from stress fractures by the presence of a well-defined T1 hypointense lesion at the fracture site completely replacing the marrow fat, ill-defined fracture line due to the tumour tissue eroding the bone trabeculae, endosteal scalloping, presence of soft tissue component and presence of necrotic component in enhancing soft tissues. In stress fractures, oedema tends to be ill-defined and irregular with interspersed marrow fat on T1 images. Pathological fractures are common in metaphysis, whereas stress fractures frequently involve diaphysis. In adults, an avulsion fracture of lesser trochanter is considered pathological unless proved otherwise.^1,40,41^

### Pathologies mimicking atraumatic femoral fractures

#### Osteoid osteoma

**Figure 12.**
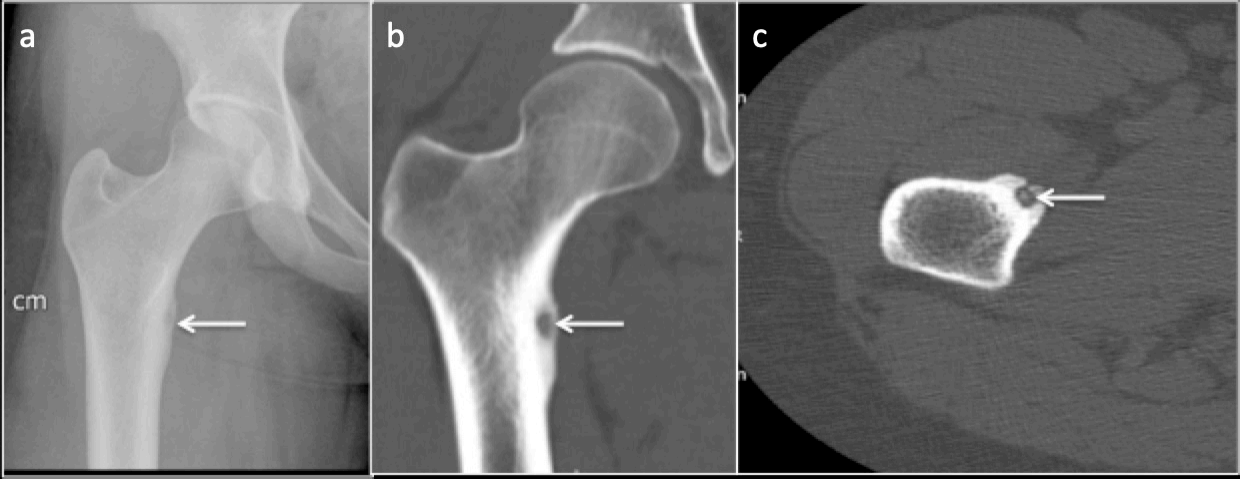
Patient presenting with hip pain. (a) Radiograph demonstrating central lucency (arrow) with surrounding sclerosis and cortical thickening along the medial cortex of proximal femur resembling a fatigue fracture. (**b**) Coronal and (c) Axial CT images demonstrating central nidus and surrounding sclerosis and cortical thickening indicating that the lesion is osteoid osteoma.

It is a benign osteoblastic tumour and the femur is most commonly affected.^42^ Usually cortically based, it appears as a focal sclerotic area with cortical thickening with a central nidus appearing as central lucency, often mimicking stress fracture. A stress fracture appears as a linear infraction in the centre of an area of surrounding cortical thickening osteoid osteoma conversely appears as a round nidus.^43^ On follow-up radiograph, the size of the stress fracture may reduce, whereas osteoid osteoma will remain stable. MRI may demonstrate marrow oedema and linear fracture line if present, in stress fracture. In osteoid osteoma of the femoral neck, MRI may demonstrate a half-moon shaped oedema pattern with the base along the cortex of the femoral neck referred to as “half-moon sign”.^44^ CT remains the investigation of choice to demonstrate the nidus in the osteoid osteoma^45^ and useful to differentiate it from a stress fracture. (Figure 12) On scintigraphy, a stress fracture shows intense tracer uptake of linear morphology, osteoid osteoma, on the other hand, displays intense central tracer uptake representing nidus with the surrounding area of moderate tracer uptake donating areas of increased osteoblastic activity, overall known as the “double-density” sign^44^ (Table 5).

**Table 5.** Differentiating stress fracture from osteoid osteoma

	Stress fracture	Osteoid osteoma
Radiograph	Linear infraction in the centre of cortical thickening	Round nidus in the centre of cortical thickening
Follow-up radiograph	May resolve or reduce in size	Stable
MRI	Marrow oedema with or without fracture line	Marrow oedema, half-moon sign
CT	Linear fracture line with surrounding sclerosis	Central nidus with surrounding sclerosis
Scintigraphy	Linear uptake	Double density sign

### Osteomyelitis

**Figure 13.**
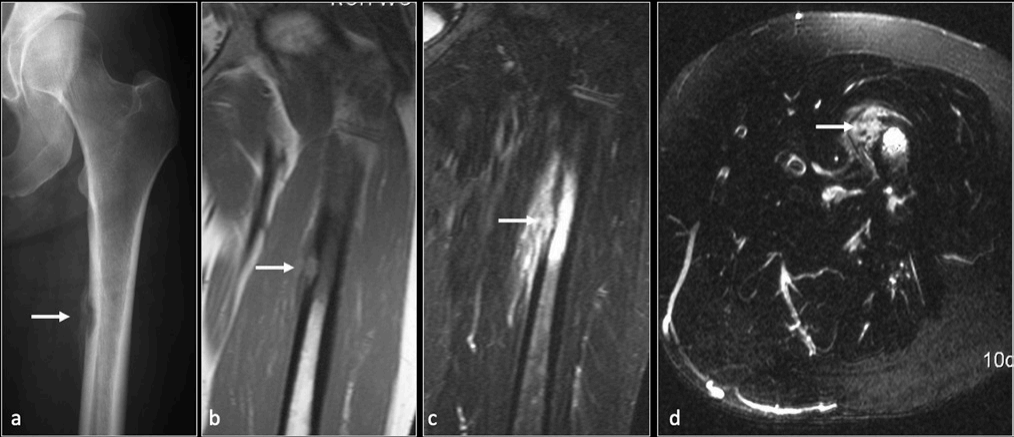
Osteomyelitis with cortical abscess femur. Anteroposterior (**a**) radiograph of the proximal femur showing lucent lesion in the medial cortex (arrow) with adjacent periosteal reaction. Coronal T1(b), coronal and axial STIR (**c, d**) MRI images demonstrating intracortical abscess (arrow) with extensive marrow and adjacent soft tissue oedema. STIR, short tau inversion recovery.

Osteomyelitis with an intracortical abscess may present with similar clinical features. Radiographs may not be helpful in differentiating osteomyelitis from a stress fracture. On MR, osteomyelitis, compared to a stress fracture, demonstrates more extensive marrow and surrounding soft tissue oedema, and an intraosseous abscess may be seen occasionally^46^ (Figure 13).

### Approach to diagnosis of atraumatic femoral fractures

After diligent attention to the patient’s demographics including age, sex, clinical and medication history, blood investigations such as blood bone profile and vitamin D levels may be needed depending on history and imaging findings.

Imaging-wise, a plain radiograph remains the primary imaging investigation, followed by further imaging with CT, MRI or nuclear scans as required can be used for further confirmation and characterisation.

## Conclusion

Atraumatic femoral fractures are an important cause of hip or thigh pain and frequently encountered in clinical practice. Although radiographs are the first investigations to be performed to confirm the diagnosis or to rule out differentials, they are not reliable, and further imaging with MRI should be considered if there is strong clinical suspicion. Some fractures such as atypical femoral fractures are often bilateral, and imaging of the contralateral side is essential. Radiologists should be aware of the nomenclature, imaging findings for timely diagnosis and appropriate communication to aid the management and to prevent further complications.

## References

[1] Marshall RA, Mandell JC, Weaver MJ, Ferrone M, Sodickson A, Khurana B. Imaging features and management of stress, atypical, and pathologic fractures. Radiographics 2018; 38: 2173–92. doi: 10.1148/rg.2018180073 30422769

[2] Pentecost RL, Murray RA, Brindley HH. Fatigue, insufficiency, and pathologic fractures. JAMA 1964; 187: 1001-4. doi: 10.1001/jama.1964.03060260029006 14102934

[3] Anderson MW, Greenspan A. Stress fractures. Radiology 1996; 199: 1–12. doi: 10.1148/radiology.199.1.8633129 8633129

[4] Wagner D, Ossendorf C, Gruszka D, Hofmann A, Rommens PM. Fragility fractures of the sacrum: how to identify and when to treat surgically? Eur J Trauma Emerg Surg 2015; 41: 349–62. doi: 10.1007/s00068-015-0530-z 26038048 PMC4523697

[5] Belthur MV, Birchansky SB, Verdugo AA, Mason EO, Hulten KG, Kaplan SL, et al. Pathologic fractures in children with acute Staphylococcus aureus osteomyelitis. J Bone Joint Surg Am 2012; 94: 34–42. doi: 10.2106/JBJS.J.01915 22218380

[6] Rohena-Quinquilla IR, Rohena-Quinquilla FJ, Scully WF, Evanson JRL. Femoral neck stress injuries: analysis of 156 cases in a U.S. military population and proposal of a new MRI classification system. AJR Am J Roentgenol 2018; 210: 601–7. doi: 10.2214/AJR.17.18639 29336599

[7] Tins BJ, Garton M, Cassar-Pullicino VN, Tyrrell PNM, Lalam R, Singh J. Stress fracture of the pelvis and lower limbs including atypical femoral fractures—a review. Insights imaging. 2015; 6: 97–110. 10.1007/s13244-014-0371-z PMC4330230 25448537

[8] Matheson GO, Clement DB, McKenzie DC, Taunton JE, Lloyd-Smith DR, MacIntyre JG. Stress fractures in athletes. A study of 320 cases. Am J Sports Med 1987; 15: 46–58. doi: 10.1177/036354658701500107 3812860

[9] Niva MH, Kiuru MJ, Haataja R, Pihlajamäki HK. Fatigue injuries of the femur. J Bone Joint Surg Br 2005; 87-B: 1385–90. doi: 10.1302/0301-620X.87B10.16666 16189313

[10] Hwang B, Fredericson M, Chung CB, Beaulieu CF, Gold GE. Mri findings of femoral diaphyseal stress injuries in athletes. AJR Am J Roentgenol 2005; 185: 166–73. doi: 10.2214/ajr.185.1.01850166 15972418

[11] Fredericson M, Un Jang K, Bergman G, Gold G. Femoral diaphyseal stress fractures: results of a systematic bone scan and magnetic resonance imaging evaluation in 25 runners. Physical Therapy in Sport 2004; 5: 188–93. doi: 10.1016/j.ptsp.2004.05.004

[12] MichaelE M. The? gray cortex?: an early sign of stress fracture. Skeletal Radiol 1995; 24. 10.1007/BF00228923 7610412

[13] Wright AA, Hegedus EJ, Lenchik L, Kuhn KJ, Santiago L, Smoliga JM. Diagnostic accuracy of various imaging modalities for suspected lower extremity stress fractures: a systematic review with evidence-based recommendations for clinical practice. Am J Sports Med 2016; 44: 255–63. doi: 10.1177/0363546515574066 25805712

[14] Arendt EA, Griffiths HJ. The use of Mr imaging in the assessment and clinical management of stress reactions of bone in high-performance athletes. Clin Sports Med 1997; 16: 291–306. doi: 10.1016/S0278-5919(05)70023-5 9238311

[15] Hagino H, Okano T, Teshima R, Nishi T, Yamamoto K. Insufficiency fracture of the femoral head in patients with severe osteoporosis--report of 2 cases. Acta Orthop Scand 1999; 70: 87–9. doi: 10.3109/17453679909000966 10191757

[16] Rafii M, Mitnick H, Klug J, Firooznia H. Insufficiency fracture of the femoral head: MR imaging in three patients. AJR Am J Roentgenol 1997; 168: 159–63. doi: 10.2214/ajr.168.1.8976940 8976940

[17] Yamamoto T. Subchondral insufficiency fractures of the femoral head. Clin Orthop Surg 2012; 4: 173. doi: 10.4055/cios.2012.4.3.173 22949947 PMC3425646

[18] Yamamoto T, Nakashima Y, Shuto T, Jingushi S, Iwamoto Y. Subchondral insufficiency fracture of the femoral head in younger adults. Skeletal Radiol 2007; 36 Suppl 1: 38–42. doi: 10.1007/s00256-006-0178-1 16944140

[19] Song WS, Yoo JJ, Koo K-H, Yoon KS, Kim Y-M, Kim HJ. Subchondral fatigue fracture of the femoral head in military recruits. J Bone Joint Surg Am 2004; 86: 1917–24. doi: 10.2106/00004623-200409000-00009 15342753

[20] Iwasaki K, Yamamoto T, Nakashima Y, Mawatari T, Motomura G, Ikemura S, et al. Subchondral insufficiency fracture of the femoral head after liver transplantation. Skeletal Radiol 2009; 38: 925–8. doi: 10.1007/s00256-009-0706-x 19418050

[21] Miyanishi K, Hara T, Hamada T, Maekawa M, Tsurusaki S, Moro-oka T-aki, et al. Co-Occurrence of subchondral insufficiency fracture of the femoral head and contralateral femoral neck fracture in a rheumatic patient receiving steroid treatment. Mod Rheumatol 2008; 18: 619–22. doi: 10.3109/s10165-008-0093-5 18584289

[22] Watanabe W, Itoi E, Yamada S. Early MRI findings of rapidly destructive coxarthrosis. Skeletal Radiol 2002; 31: 35–8. doi: 10.1007/s00256-001-0445-0 11807591

[23] Yamamoto T, Schneider R, Bullough PG. Subchondral insufficiency fracture of the femoral head: histopathologic correlation with MRI. Skeletal Radiol 2001; 30: 247–54. doi: 10.1007/s002560100348 11407715

[24] Miyanishi K, Hara T, Kaminomachi S, Maeda H, Watanabe H, Torisu T. Contrast-Enhanced MR imaging of subchondral insufficiency fracture of the femoral head: a preliminary comparison with that of osteonecrosis of the femoral head. Arch Orthop Trauma Surg 2009; 129: 583–9. doi: 10.1007/s00402-008-0642-6 18542974

[25] Ikemura S, Yamamoto T, Motomura G, Nakashima Y, Mawatari T, Iwamoto Y. Mri evaluation of collapsed femoral heads in patients 60 years old or older: differentiation of subchondral insufficiency fracture from osteonecrosis of the femoral head. AJR Am J Roentgenol 2010; 195: W63–8. doi: 10.2214/AJR.09.3271 20566783

[26] Iwasaki K, Yamamoto T, Motomura G, Ikemura S, Mawatari T, Nakashima Y, et al. Prognostic factors associated with a subchondral insufficiency fracture of the femoral head. Br J Radiol 2012; 85: 214–8. doi: 10.1259/bjr/44936440 21159802 PMC3473999

[27] Malizos KN, Zibis AH, Dailiana Z, Hantes M, Karachalios T, Karahalios T, Karantanas AH, et al. Mr imaging findings in transient osteoporosis of the hip. Eur J Radiol 2004; 50: 238–44. doi: 10.1016/j.ejrad.2004.01.020 15145483

[28] Chang CY, Rosenthal DI, Mitchell DM, Handa A, Kattapuram SV, Huang AJ. Imaging findings of metabolic bone disease. Radiographics 2016; 36: 1871–87. doi: 10.1148/rg.2016160004 27726750

[29] McKenna MJ, Kleerekoper M, Ellis BI, Rao DS, Parfitt AM, Frame B. Atypical insufficiency fractures confused with Looser zones of osteomalacia. Bone 1987; 8: 71–8. doi: 10.1016/8756-3282(87)90073-1 3593610

[30] McKenna MJ, Heffernan E, Hurson C, McKiernan FE. Clinician approach to diagnosis of stress fractures including Bisphosphonate-associated fractures. QJM 2014; 107: 99–105. doi: 10.1093/qjmed/hct192 24106312

[31] Smith SE, Murphey MD, Motamedi K, Mulligan ME, Resnik CS, Gannon FH. From the Archives of the AFIP. radiologic spectrum of Paget disease of bone and its complications with pathologic correlation. Radiographics 2002; 22: 1191–216. doi: 10.1148/radiographics.22.5.g02se281191 12235348

[32] Cortis K, Micallef K, Mizzi A. Imaging Paget's disease of bone--from head to toe. Clin Radiol 2011; 66: 662–72. doi: 10.1016/j.crad.2010.12.016 21524738

[33] Yoon RS, Hwang JS, Beebe KS. Long-Term bisphosphonate usage and subtrochanteric insufficiency fractures: a cause for concern? J Bone Joint Surg Br 2011; 93: 1289–95. doi: 10.1302/0301-620X.93B10.26924 21969423

[34] Shane E, Burr D, Abrahamsen B, Adler RA, Brown TD, Cheung AM, et al. Atypical subtrochanteric and diaphyseal femoral fractures: second report of a task force of the American Society for bone and mineral research. J Bone Miner Res 2014; 29: 1–23. doi: 10.1002/jbmr.1998 23712442

[35] Larsen MS, Schmal H. The enigma of atypical femoral fractures: a summary of current knowledge. EFORT Open Rev 2018; 3: 494–500. doi: 10.1302/2058-5241.3.170070 30305933 PMC6174857

[36] Schilcher J, Sandberg O, Isaksson H, Aspenberg P. Histology of 8 atypical femoral fractures: remodeling but no healing. Acta Orthop 2014; 85: 280–6. doi: 10.3109/17453674.2014.916488 24786905 PMC4062796

[37] Taormina DP, Marcano AI, Karia R, Egol KA, Tejwani NC. Symptomatic atypical femoral fractures are related to underlying hip geometry. Bone 2014; 63: 1–6. doi: 10.1016/j.bone.2014.02.006 24565751

[38] Kilcoyne A, Heffernan EJ. Atypical proximal femoral fractures in patients with Paget disease receiving bisphosphonate therapy. AJR Am J Roentgenol 2011; 197: W196–7. doi: 10.2214/AJR.10.6343 21700989

[39] Kwek EBK, Goh SK, Koh JSB, Png MA, Howe TS. An emerging pattern of subtrochanteric stress fractures: a long-term complication of alendronate therapy? Injury 2008; 39: 224–31. doi: 10.1016/j.injury.2007.08.036 18222447

[40] Pauleit D, Sommer T, Textor J, Flacke S, Hasan C, Steuer K, et al. Mri diagnosis in longitudinal stress fractures: differential diagnosis of Ewing sarcoma. Rofo 1999; 170: 28–34. doi: 10.1055/s-2007-1011003 10071641

[41] Fayad LM, Kawamoto S, Kamel IR, Bluemke DA, Eng J, Frassica FJ, et al. Distinction of long bone stress fractures from pathologic fractures on cross-sectional imaging: how successful are we? AJR Am J Roentgenol 2005; 185: 915–24. doi: 10.2214/AJR.04.0950 16177409

[42] Resnick D, Kyrriakos M. Greenway GD Tumors and tumor-like lesions of bone: imaging and pathology of specific lesions. In: Resnick D, Kransdorf M. J, eds.Bone and joint imaging. 3rd ed. Philadelphia, PA: Saunders; 2005. pp. 1120–30.

[43] Chai JW, Hong SH, Choi J-Y, Koh YH, Lee JW, Choi J-A, et al. Radiologic diagnosis of osteoid osteoma: from simple to challenging findings. Radiographics 2010; 30: 737–49. doi: 10.1148/rg.303095120 20462991

[44] Klontzas ME, Zibis AH, Karantanas AH. Osteoid osteoma of the femoral neck: use of the Half-Moon sign in MRI diagnosis. AJR Am J Roentgenol 2015; 205: 353–7. doi: 10.2214/AJR.14.13689 26204287

[45] Assoun J, Richardi G, Railhac JJ, Baunin C, Fajadet P, Giron J, et al. Osteoid osteoma: MR imaging versus CT. Radiology 1994; 191: 217–23. doi: 10.1148/radiology.191.1.8134575 8134575

[46] Jaimes C, Jimenez M, Shabshin N, Laor T, Jaramillo D. Taking the stress out of evaluating stress injuries in children. Radiographics 2012; 32: 537–55. doi: 10.1148/rg.322115022 22411948

