# Supplementary material for: Atraumatic fractures of the femur
Source: Br J Radiol. 2021 Mar 18;94(1121):20201457. doi: 10.1259/bjr.20201457 (PMC8506166; doi:10.1259/bjr.20201457)
Supplement: Supplementary Figure 2. [file bjr.20201457.suppl-02.docx]

**Flow chart 2** Pathogenesis of Atypical Femoral Fracture
